# Sex differences in the association between childhood maltreatment and cardiovascular disease in the UK Biobank

**DOI:** 10.1136/heartjnl-2019-316320

**Published:** 2020-07-14

**Authors:** Ana Luiza Gonçalves Soares, Gemma Hammerton, Laura D Howe, Janet Rich-Edwards, Sarah Halligan, Abigail Fraser

**Affiliations:** 1 Population Health Sciences, Bristol Medical School, University of Bristol, Bristol, UK; 2 MRC Integrative Epidemiology Unit, University of Bristol, Bristol, UK; 3 Department of Medicine, Harvard Medical School, Boston, Massachusetts, USA; 4 Brigham and Women’s Hospital and Department of Epidemiology, Harvard University T H Chan School of Public Health, Boston, Massachusetts, USA; 5 Department of Psychology, University of Bath, Bath, UK; 6 Department of Psychiatry and Mental Health, University of Cape Town, Cape Town, South Africa

**Keywords:** cardiac risk factors and prevention, heart disease, epidemiology

## Abstract

**Objectives:**

To assess and compare associations between childhood maltreatment and cardiovascular disease (CVD) in men and women in the UK. In secondary analyses, we also explored possible age differences and associations with early onset CVD (<50 years).

**Methods:**

We included 157 311 participants from the UK Biobank who had information on physical, sexual or emotional abuse, emotional or physical neglect. CVD outcomes were defined as any CVD, hypertensive disease, ischaemic heart disease (IHD) and cerebrovascular disease. These were extracted from self-report, blood pressure measurements, hospital register and death register. The associations between maltreatment and CVD were assessed using Poisson regression with robust variance to estimate risk ratios, stratified by sex and adjusted for socioeconomic and demographic factors.

**Results:**

All types of maltreatment were associated with increased risk of CVD and IHD in both sexes. Additionally, in women all types of maltreatment were associated with higher risk of hypertensive disease, and all, except emotional neglect, were associated with cerebrovascular disease. In men, all but sexual abuse, were associated with higher risk of hypertensive disease, and all, except physical and sexual abuse, were associated with cerebrovascular disease. Associations were generally stronger in women, and individuals who were younger at baseline had stronger associations of childhood maltreatment with any CVD and IHD, but age differences were less evident when only early onset CVD was considered.

**Conclusions:**

Childhood maltreatment was consistently associated with CVD and stronger associations were generally observed in women and seemed to be stronger for early onset CVD.

## Introduction

Childhood maltreatment has been robustly associated with increased risk for many adverse mental and physical health outcomes, including cardiovascular diseases (CVD).^1–4^ A recent systematic review showed that childhood maltreatment, which comprises physical, sexual and emotional abuse and neglect, was associated with CVD – incorporating myocardial infarction (MI), stroke, ischaemic heart disease (IHD) and coronary heart disease (CHD) – in more than 90% of the 24 studies that included CVD endpoints.^5^

Men are more likely to report physical abuse, while women are more likely to report sexual abuse, emotional abuse and neglect, as well as a higher number of adversities.^1 2 4 6^ Sex differences in CVD also exist.^7^ Men are more likely to develop CHD as their first event, while women are more likely to have cerebrovascular disease or heart failure,^8^ later onset CVD and lower mortality.^9 10^ Some evidence suggests that women are more vulnerable to the consequences of psychosocial stress,^1 2^ but investigation of sex differences in the association between childhood maltreatment and CVD has been limited, and the evidence to date shows no consistent pattern.^2 5 11 12^ Understanding such sex differences might help to identify sex-specific protective or maladaptive mechanisms by which childhood maltreatment might affect CVD.

Age is the most important determinant of cardiovascular health and is plausibly related to the risk and/or recall of childhood maltreatment. A recent study assessing the association of psychiatric stress-related disorders and risk of CVD showed a stronger association in younger ages,^13^ suggesting that the effects of stress on CVD might vary with age. To the best of our knowledge, no studies have assessed potential modification by age in the association between childhood maltreatment and CVD.

We use data from a large cohort in the UK to assess the association between childhood maltreatment and CVD, and to examine sex differences in this relationship. Given the wide age range of the participants (40–69 years at baseline) and the possibility of age differences in the associations, we also tested whether the associations were stable across different age groups. Finally, we limited analyses to early onset CVD (<50 years of age) in all age groups to ensure comparability regarding the ‘time at risk’.

## Methods

The UK Biobank includes 502 524 participants aged 40–69 years at baseline, recruited between 2006 and 2010.^14 15^ In 2016, 339 092 participants who provided an email address were invited to complete an online mental health questionnaire, which included information on childhood maltreatment.^14^ From the 157 366 participants who completed the questionnaire (46% of those emailed),^14^ 157 311 responded to at least one question about childhood maltreatment, comprising the sample of this study.

Childhood maltreatment (physical, sexual and emotional abuse, emotional and physical neglect) was assessed using the Childhood Trauma Screener.^16^ The questions and cut-off points used are presented in online supplementary eTable 1.

10.1136/heartjnl-2019-316320.supp1 Supplementary data

CVD outcomes include self-reported diagnosis of vascular/heart problems, self-reported use of blood pressure medication and measured blood pressure, all assessed at baseline, as well as hospital and death registers. Hospital registers cover the period between 1997 until March 2017, and death registers cover the period between May 2006 and February 2018. Details on how CVD outcomes were assessed are presented in online supplementary material, and the timeline for the data is illustrated in online supplementary eFigure 1. Considering a previous study found stronger associations for self-reported CVD than medically verified cases,^3^ in sensitivity analyses only medical records and measured blood pressure were used to define the outcomes.

Potential confounders were selected a priori based on their known or plausible effects on childhood maltreatment and CVD. Age, ethnicity, Townsend deprivation index, maternal smoking around birth and family history of CVD were used as confounders. The UK Biobank lacks childhood socioeconomic measures, and considering smoking is strongly socioeconomically patterned, we used maternal smoking as a proxy of childhood socioeconomic position (SEP)^17^ and Townsend deprivation index, as a proxy for early-life SEP due to tracking. Other factors related to CVD were assessed to describe the sample among the UK Biobank participants who responded and did not respond to the childhood maltreatment questionnaire. All confounders and covariates were assessed at baseline and more details are available in online supplementary material.

## Statistical analysis

All analyses were conducted using Stata version 15.1 (Statacorp, College Station, TX). Childhood maltreatment types were assessed individually and using a summary score, ranging from 0 (no experience of maltreatment) to 4+ (experience of four or five types of maltreatment). We summarised all covariates and outcomes in participants included (with information on childhood maltreatment) and not included in the analysis.

As the outcome was frequent, associations between childhood maltreatment and CVD were assessed using Poisson regression with robust variance to estimate risk ratios (RRs), unadjusted and adjusted for the confounders defined above. The analyses were performed stratified by sex, and evidence for sex differences were determined by comparing results between men and women and computing statistical tests for interactions between sex and each type of maltreatment.

We used stabilised inverse probability weights (IPW) to reduce selection bias from missing data due to non-response to the online questionnaire.^18 19^ More details about the propensity score and weighting procedure are described in online supplementary material.

In order to investigate whether the associations between childhood maltreatment and CVD differed by age, as secondary analysis we estimated age-stratified (40–49y, 50–59y and 60+years) RRs. We computed statistical tests for interactions between age groups and each type of maltreatment. We also repeated analyses restricting the outcome to early onset CVD, defined as any CVD occurring before 50 years, to ensure comparable ‘time at risk’ for all age groups. Age of CVD onset was based on baseline self-reported age of diagnosis of heart attack, angina, stroke or high blood pressure. Similarly, statistical tests for interactions between early onset CVD and each type of maltreatment were computed.

## Results

Responders to the UK Biobank online questionnaire with valid information for childhood maltreatment were more likely to be female and white, to have lower age, to experience less deprivation, to have higher education, lower prevalence of maternal smoking around birth, current smoking and depression, and higher frequency of alcohol intake than non-responders to the online questionnaire (table 1). Responders were also more likely to have more favourable cardiovascular health (lower systolic and diastolic blood pressures, lower body mass index (BMI), and lower use of blood pressure and cholesterol-lowering medications), and lower occurrence of CVD than non-responders.

**Table 1:** Comparison of responders and non-responders to the mental health online questionnaire containing childhood maltreatment in the UK Biobank

	Men		Women	
	Non-responders n=160 894 (70.2%)	Responders* n=68 240 (29.8%)	Non-responders n=184 240 (67.4%)	Responders* n=89 071 (32.6%)
Continuous variables: mean (SD)				
Age (years)	56.8 (8.4)	56.6 (7.8)	56.8 (8.1)	55.5 (7.7)
SBP (mmHg)	141.3 (17.8)	140.0 (16.8)	136.3 (19.5)	133.4 (18.4)
DBP (mmHg)	84.2 (10.1)	83.8 (9.8)	81.0 (10.1)	80.1 (9.8)
BMI (kg/m^2^)	28.0 (4.3)	27.3 (4.0)	27.4 (5.3)	26.4 (4.9)
Categorical variables: %				
Ethnicity (white)	93.5	97.1	93.4	97.1
Townsend deprivation index				
First quintile (20% least deprived)	18.7	23.5	19.0	22.1
Fifth quintile (20% most deprived)	22.9	15.2	21.2	15.9
Qualifications				
None of the below	22.0	7.2	22.2	6.8
Other/NVQ/CSE	20.8	15.5	17.3	12.8
O-level	19.3	17.4	24.4	21.9
A-level	9.6	12.3	10.7	14.5
College/university degree	28.4	47.5	25.4	44.1
Maternal smoking				
No	60.2	62.0	62.6	63.9
Yes	26.5	25.8	25.1	25.2
Do not know	13.4	12.2	12.3	10.9
Smoking status				
Never smoker	34.4	35.5	45.2	43.9
Former smoker	51.4	55.9	44.5	49.9
Current smoker	14.3	8.6	10.3	6.2
Alcohol intake				
Daily or almost daily	24.0	28.6	14.6	19.1
3–4 x/week	24.9	28.7	18.8	23.9
1–2 x/week	26.6	24.2	25.9	25.4
1–3 x/month	9.1	8.5	13.1	12.9
Special occasions only	8.1	5.6	16.6	12.0
Never	7.2	4.4	11.0	6.5
Depression	27.2	25.6	42.4	40.2
Use of blood pressure medication	26.6	20.5	20.2	12.6
Use of cholesterol-lowering medication	24.5	19.7	14.7	8.8
Family history of CVD	70.7	72.5	77.4	77.7
Any CVD	63.2	54.3	52.0	40.3
Hypertensive disease	51.9	43.4	40.1	29.4
Ischaemic heart disease	15.6	10.0	7.1	3.6
Cerebrovascular disease	5.1	2.5	3.3	1.4
Early onset	12.6	10.0	9.4	7.1

All P-values for comparisons between non-responders and responders were <0.001, except for family history of CVD in women, which was 0.022.

*From the responders to the childhood maltreatment questionnaire, 98.4% of men and 97.1% of women responded to all questions on childhood maltreatment.

A-level, advanced level or equivalent; BMI, Body mass index; CSE, certificate of secondary education or equivalent; CVD, cardiovascular disease; DBP, diastolic blood pressure; HND, higher national diploma of equivalent; NVQ, national vocational qualification; O-level, ordinary level; SBP, systolic blood pressure.

**Figure 1:**
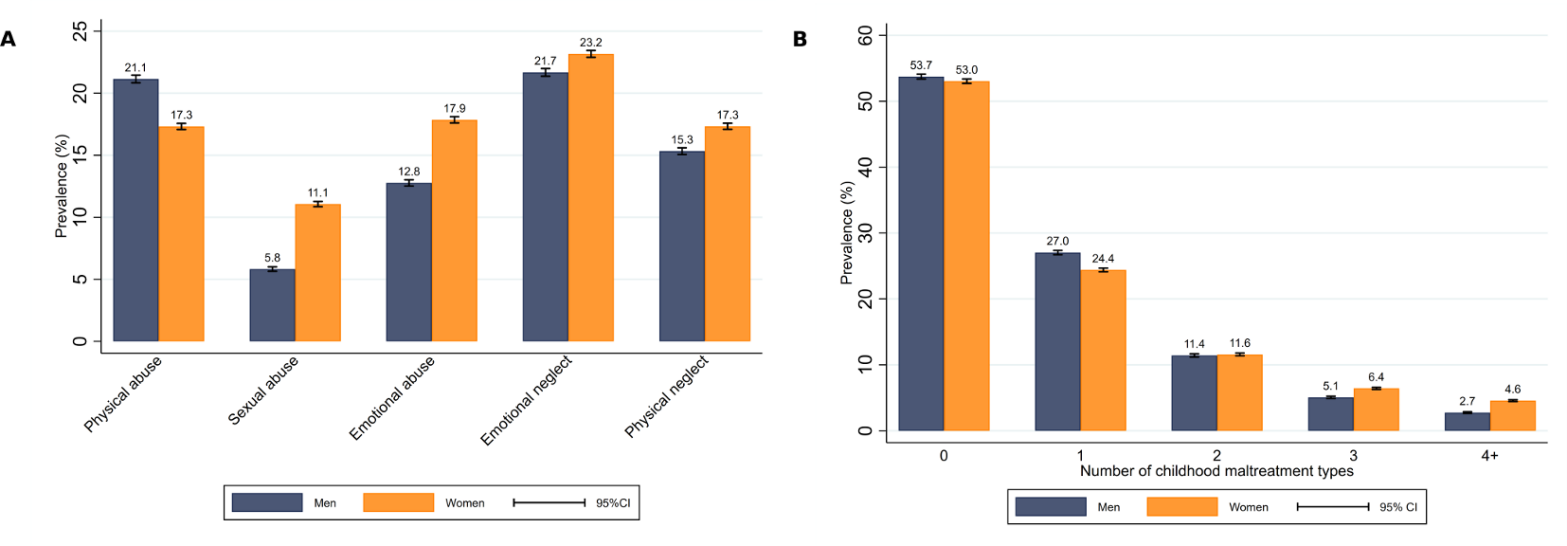
Prevalence of types of childhood maltreatment (A) and number of childhood maltreatment types experienced (B) in men and women.

Emotional neglect was the most common type of childhood maltreatment (22.5%), followed by physical abuse in men (21.1%) and emotional abuse in women (17.9%) (figure 1A). All types of maltreatment were more prevalent in women except for physical abuse, which was more prevalent in men. Women were also more likely to experience a higher number of types of childhood maltreatment: 4.6% of women experienced four or more types of maltreatment, while 2.7% of men experienced the same number (figure 1B). The prevalence of childhood maltreatment decreased with increasing age, except for sexual abuse in men, which did not vary, and physical neglect, which increased across age categories (see online supplementary eFigure 2a). Younger participants also reported a higher number of maltreatment types, especially women (see online supplementary eFigure 2b). The occurrence of any CVD was 54.3% in men and 40.3% in women, and all CVD types were more common in men (table 1, online supplementary eTable 2). When only medical records and measured blood pressure were considered, the occurrence of CVD was lower, but the same sex and age patterns were observed (see online supplementary eTable 2).

**Figure 2:**
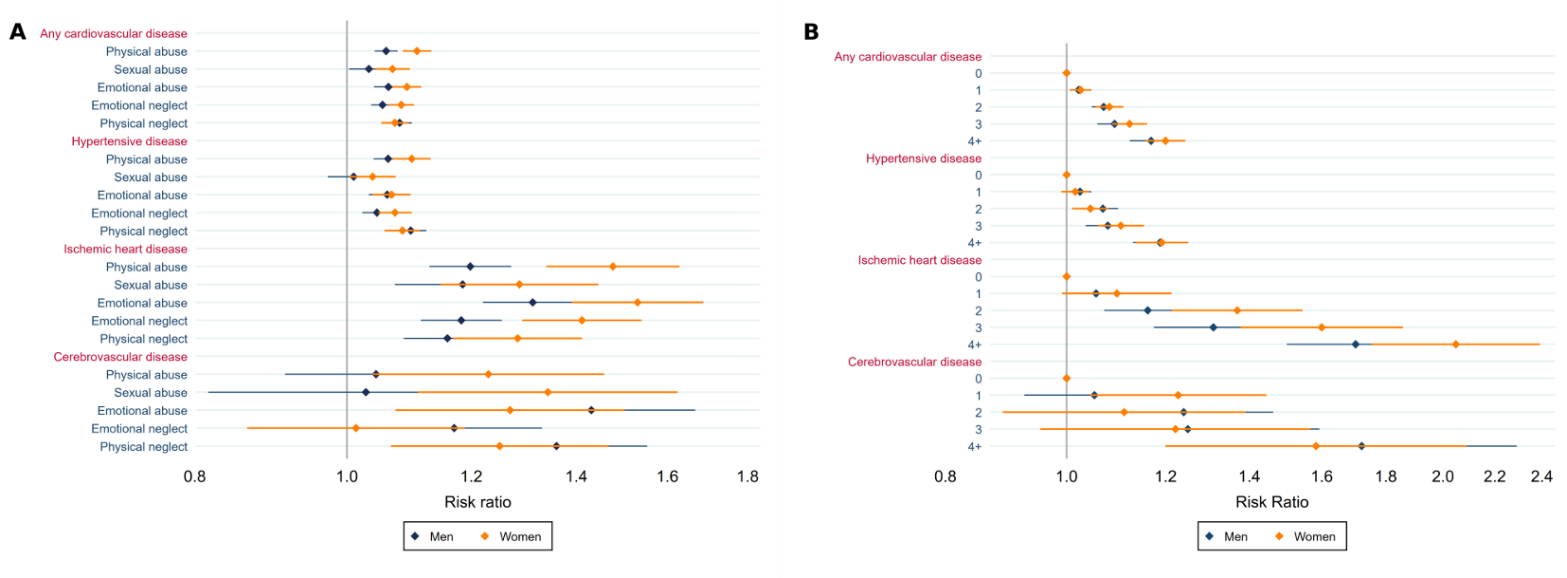
Adjusted association (risk ratios and 95% CIs) of types of childhood maltreatment (A) and number of childhood maltreatment types experienced (B) with cardiovascular disease in men and women.

All types of maltreatment were associated with a greater risk of any CVD and IHD in both sexes, even when adjusting for potential confounders (figure 2A). Additionally, in women, all types of maltreatment were associated with a greater risk of hypertensive disease and all, except emotional neglect, were associated with a greater risk of cerebrovascular disease. In men, all types of maltreatment but sexual abuse were also associated with hypertensive disease and all, except physical and sexual abuse, were associated with a greater risk of cerebrovascular disease. Associations between childhood maltreatment and CVD outcomes were generally stronger in women (figure 2A; online supplementary eTable 3). For instance, the association between physical abuse and IHD was RR=1.48 (95% CI 1.34 to 1.63) in women and RR=1.20 (95% CI 1.13 to 1.27) in men (P-value for sex interaction <0.001). Unadjusted and adjusted associations, as well as statistical tests for sex interactions, are detailed in online supplementary eTable 3. Similar results were obtained when only medical records and measured blood pressure were used to define CVD (see online supplementary eTable 4).

A dose-response effect was observed such that a higher number of maltreatment types was associated with higher risk of CVD (figure 2B). The only sex difference observed was for a stronger association between the number of types of childhood maltreatment and IHD in women. Both unadjusted and adjusted associations are presented in online supplementary eTable 5.

**Figure 3:**
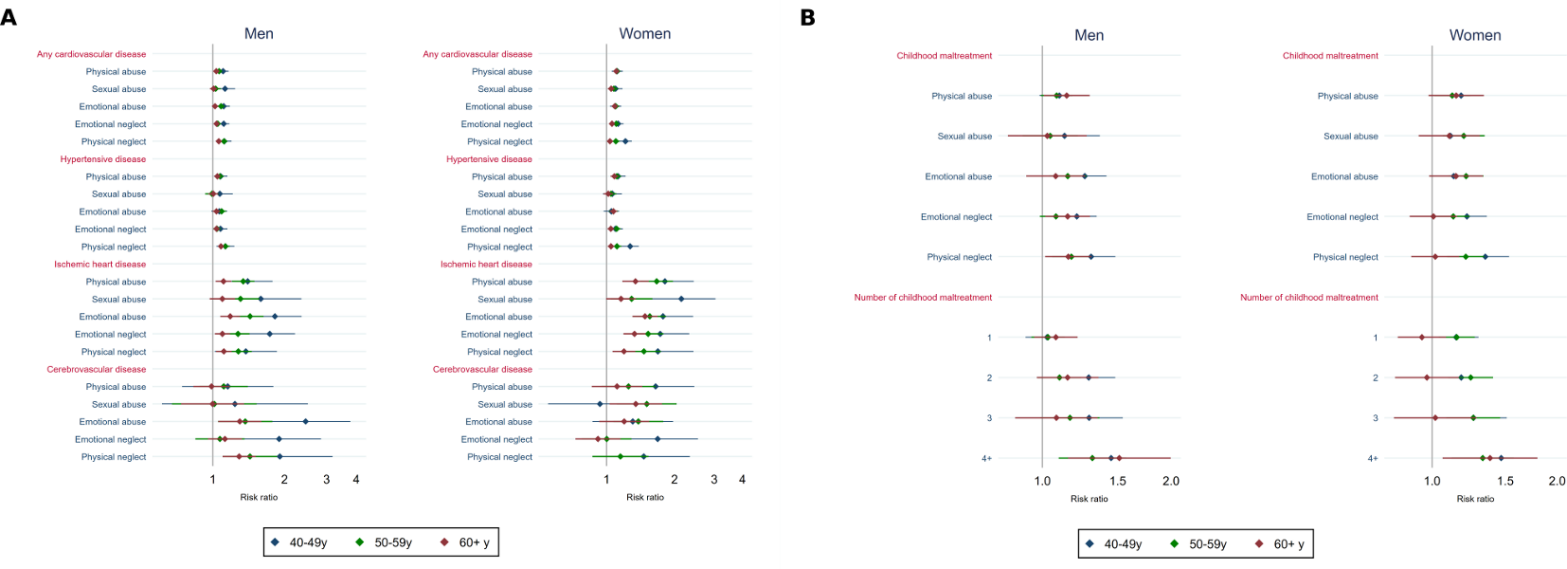
Adjusted associations (risk ratios and 95% CIs) of types of childhood maltreatment (A) with cardiovascular disease and (B) with early onset cardiovascular disease, according to age groups, in men and women.

In age-stratified analyses, associations of childhood maltreatment (individually or summed into a score) with any CVD and IHD were stronger in younger men than in older men (figure 3A, online supplementary eFigure 3, eTables 6 and 7). The RR for the association between emotional abuse and IHD, for example, was 1.82 (95% CI 1.41 to 2.36) in men aged 40–49y, 1.43 (95% CI 1.26 to 1.63) in men aged 50–59y and 1.18 (95% CI 1.08 to 1.30) in those aged 60y or more. A similar pattern was observed in women for the associations of emotional and physical neglect with any CVD and hypertensive disease, of physical and sexual abuse and emotional and physical neglect with IHD, between emotional neglect and cerebrovascular disease, and of a higher number of maltreatment types with any CVD, hypertensive disease and IHD. When analyses were limited to early onset CVD, associations with maltreatment generally remained, but a stronger association in younger individuals was observed only for physical neglect in men, and for emotional and physical neglect in women (figure 3B, online supplementary eTable 8). We therefore directly compared associations of childhood maltreatment with early and later onset CVD among participants aged >50 at baseline. This suggested that associations with early onset CVD were of greater magnitude, especially in men, though CIs overlap (see online supplementary eFigure 4).

## Discussion

Overall, all five types of maltreatment were associated with higher risk of CVD. There was some evidence of stronger associations in women and younger individuals, but age differences were less evident when only early onset CVD was considered.

A recent systematic review showed a wide variation on the prevalence of self-reported childhood maltreatment, which reflects methodological differences, including data collection methods and maltreatment definitions, in addition to geographical and sex differences.^6^ In the UK, the prevalence of physical abuse ranged from 3.6% to 32.6%, sexual abuse from 0.7% to 27.8%, emotional abuse from 4.0% to 66.7% and neglect from 5.6% to 77.8%.^6^ Our estimates of childhood maltreatment lie between those ranges.

Most studies showing that childhood maltreatment is associated with increased CVD are based on self-reported diagnosis of CVD.^5^ We confirmed these findings using combined self-reported and medically confirmed cases of CVD, as well as medically confirmed cases only, with similar results observed in both scenarios. As hospital records in the UK Biobank date back to 1997, cases occurring before that would have been missed without the use of self-reported information.

The magnitude of the associations with the different types of CVD was similar across the types of childhood maltreatment, and stronger associations were observed for IHD and cerebrovascular disease, especially in women. Better understanding of the pathways that link childhood maltreatment to CVD is needed. Possible mechanisms include health-related behavioural factors (i.e., smoking, overeating, physical inactivity), mental health (i.e., depression, anxiety) and biological factors (i.e., altered stress response, chronic inflammation and metabolic changes).^2 3 13 20 21^ These potential mediators are by no means mutually exclusive and may be part of the same pathways, for example, maltreatment causing depression, physical inactivity, greater adiposity and inflammation, resulting in CVD. Work to understand these different pathways is ongoing.

Few studies have explored sex differences in the association between childhood maltreatment and CVD, and the available evidence has not yielded consistent findings.^2 5 11 21^ In our study, most associations between childhood maltreatment and CVD were found in both sexes and, when sex differences were evident, women had stronger associations. Sex differences are evident in the hypothalamic-pituitary-adrenal axis dysregulation in response to stress,^22 23^ and women might be more vulnerable to the consequences of psychosocial stress, such as childhood maltreatment.^1 2^ Other risk factors for CVD, which may mediate this association, also differ between men and women and might explain some of the sex differences. A recent study showed that although systolic blood pressure, hypertension, smoking status and diabetes were associated with MI in both men and women, the associations were stronger in the latter.^24^ A better understanding on why sex differences emerge might help to inform interventions to reduce the burden of CVD in those who suffered maltreatment.

Younger individuals, especially men, had stronger associations between maltreatment and both CVD and IHD. The magnitude of the associations for all CVD events and for early onset CVD were overall similar, and some – though not all – age differences were attenuated when only early onset CVD was considered. This analysis was undertaken as participants aged 40–49 years could not have experienced a ‘late’ onset CVD event, which could be a reason for the observed effect modification by age. We then confirmed that the magnitude of associations of maltreatment with early onset CVD were usually larger than for later onset events, especially in men, though with overlapping CIs. Of note, a recent Swedish registry-based study reported that, similar to our findings, psychiatric stress-related disorders were associated with increased CVD risk, and with early onset CVD in particular.^13^ Other potential explanations for the observed differences (other than stronger associations with early onset CVD) are survival bias, age (and CVD) related bias in recall of childhood maltreatment, different aetiology of CVD with age and memory bias in age of CVD onset. Further studies assessing whether these age differences replicate in other populations and whether the risk differs according to age of CVD onset would be useful.

Studies exploring possible gene-environment interactions between childhood maltreatment and CVD would also be valuable, assessing whether genetic heritability of CVD differs in individuals who suffered and did not suffer maltreatment, and potentially whether sex also plays a role in this relationship.

## Strengths and limitations

To our knowledge, this is the largest study assessing the association between childhood maltreatment and CVD in both men and women and exploring different types of maltreatment and different types of CVD. Our sample was large enough to investigate differences in the associations by sex and the possibility of differences by age. The use of self-report in combination with medically confirmed cases of CVD is also a strength.

We acknowledge that the large sample size and the multiple tests carried out can increase type 1 error. However, we interpreted our results based on the estimates and their CIs, rather than statistical significance,^25^ and therefore we did not formally correct for multiple comparisons.

A key limitation of this study is selection bias. This arises from: the use of a cohort which had a low response rate (~5%) and is not representative of the UK population;^26^ and the use of a selected subset of participants, who differed from the original cohort. We used IPW to minimise selection bias due to responsiveness to the questionnaire in which maltreatment was reported.^27^ This resulted in a good balance of the measured covariates between those who responded and did not respond to the online questionnaire (see online supplementary eFigure 3), therefore, reducing bias due to selective participation. Of note, unweighted estimates were similar to weighted estimates, indicating that bias due to selection to the online questionnaire did not substantially affect results (results available from authors on request). However, the UK Biobank participants are less socioeconomically deprived, and more likely to be older, female and healthier than the general population, and have a lower prevalence of CVD.^26^ Furthermore, they are probably less likely to have experienced childhood maltreatment, and bias due to selective participation in the UK Biobank cannot be ruled out. This selection bias can induce collider bias, which would lead to biased estimates, in this case, negative.^28^ Therefore, estimates presented here are likely to be underestimated.

There is no gold standard to assess childhood maltreatment and both prospective and retrospective reports entail potential limitations. Like most large studies, we used self-reported retrospective measures of childhood maltreatment, which might be affected by recall bias and/or measurement error (e.g., affected by individual’s emotional state at the time of report, perceptions, interpretations or existential recollections).^29^ The alternative to self-report is police, court or other administrative records of abuse and neglect. However, only the minority of and the more severe cases are reported to authorities, whereas retrospective reports might detect more true cases.^29^ Although studies have shown modest or poor agreement between prospective and retrospective measures of childhood adversities,^29 30^ the use of retrospective measures is valid in population studies but might overestimate the association with subjectively measured outcomes (e.g., self-reported) and underestimate the association with objectively measured outcomes (e.g., physical health).^30^ Our associations were similar when compared with only medically confirmed cases of CVD; therefore, if bias due to use of retrospective measures of maltreatment is present, it is likely to have underestimated the associations.

We used maternal smoking as a proxy of childhood SEP and Townsend deprivation index as a proxy for early-life SEP, which might be insufficient to adjust for socioeconomic confounding; therefore, our associations might be biased by residual confounding. However, the magnitude of the associations was similar or slightly lower, though still evident, when we further adjusted for education (results available from authors on request), thus residual confounding is unlikely to have driven the associations observed.

## Conclusions

All types of maltreatment were associated with higher risk of CVD in both men and women, with stronger associations in the latter and in younger participants, but some age differences disappeared when only early onset CVD was considered. Interventions that ameliorate the negative effects of childhood maltreatment are needed, as well as more understanding of the pathways that link childhood maltreatment to CVD and whether they differ by sex, types of maltreatment and CVD types.

### Patient and public involvement

Details of patient and public involvement in the UK Biobank are available online (https://www.ukbiobank.ac.uk/wp-content/uploads/2011/07/Summary-EGF-consultation.pdf). No patients were directly involved in setting the research question, developing plans for recruitment, design or implementation of this study. No patients were asked to advise on interpretation or writing of the results. There are no specific plans to disseminate the results of the research to study participants, but the UK Biobank disseminates key findings from projects on its website (https://www.ukbiobank.ac.uk/published-papers; https://www.ukbiobank.ac.uk/news/).

**Key questions**

**What is already known on this subject?**

- Childhood maltreatment has been consistently associated with CVD. However, most studies assessed self-reported CVD, and few have used medically verified cases and/or investigated different types of CVD. Despite the occurrence of both childhood maltreatment and CVD differs by sex, sex differences in their association have been underexplored, and the evidence to date shows no consistent pattern.

**What might this study add?**

- This study contributes to the growing body of evidence demonstrating an association between childhood maltreatment and CVD. The magnitude of the associations with the different types of CVD was similar across all the types of childhood maltreatment, and stronger associations were observed for ischaemic heart disease and cerebrovascular disease. When associations differed by sex, stronger associations were observed in women, and results suggested stronger associations for early onset CVD (occurring before the age of 50).

**How might this impact on clinical practice?**

- Individuals who suffered maltreatment in childhood have higher risk of CVD, especially women, and might benefit from early screening and interventions to prevent cardiovascular consequences.
