# Supplementary material for: Sex differences in the association between childhood maltreatment and cardiovascular disease in the UK Biobank
Source: Heart. 2020 Jul 14;106(17):1310–6. doi: 10.1136/heartjnl-2019-316320 (PMC7476280; doi:10.1136/heartjnl-2019-316320)
Supplement: Supplementary data [file heartjnl-2019-316320supp001.pdf]

Sex differences in the association between childhood maltreatment and cardiovascular disease

ONLINE MATERIAL

Supplementary methods

Cardiovascular disease outcomes

Self-reported diagnosis of cardiovascular disease (CVD) was assessed at baseline using the question: “Has a doctor ever told you that you have had any of the following conditions?”, considering the options: heart attack, angina, stroke, high blood pressure, blood clot in the leg (deep vein thrombosis) and blood clot in the lung. Self-reported use of blood pressure medication was assessed by the question “Do you regularly take any of the following medications?”, with blood pressure medication being one of the options. Systolic blood pressure (SBP) and diastolic blood pressure (DBP) were measured twice at baseline assessment using Omron 750 IT electronic blood pressure monitor, and the mean of both measures was used. Hospital registers and death registers were coded according to the International Classification of Diseases (ICD). Hospital records date back to 1997 for England, 1998 for Wales and 1981 for Scotland, and death registers covers the period between May 2006 and February 2018.

We used as outcomes: any CVD (ICD-10 codes: I00-I99, ICD-9 codes: 390-459, self-reported diagnosis of vascular/heart problems, self-reported diagnosis of blood clot in the leg or in the lung, use of blood pressure medication, or SBP ≥140 mmHg and DBP ≥90 mmHg); hypertensive disease (ICD-10 codes: I10-I15, ICD-9 codes: 401-405, self-reported diagnosis of high blood pressure, self-reported use of blood pressure medication, or SBP ≥140 mmHg and DBP ≥90 mmHg); IHD (ICD-10 codes: I20-I25, ICD-9 codes: 410-414, or self-reported diagnosis of heart attack or angina); and cerebrovascular disease (ICD-10 codes: I60-I69, ICD-9 codes: 430-438, or self-reported diagnosis of stroke).

Confounders

Age, ethnicity, Townsend deprivation index, maternal smoking around birth and family history of CVD were assessed at baseline. Age (complete years) was calculated based on date of birth and date of attending the initial assessment centre. Ethnicity was classified as White, Mixed, Asian, Black, Chinese and Others. Townsend deprivation index was calculated based on preceding national census output areas, and each participant was assigned a score

corresponding to the output area in which their residing postcode was located; for descriptive analysis, the index was divided into quintiles. Maternal smoking around birth (yes/no) was assessed by the question: "Did your mother smoke regularly around the time when you were born?". Family history of CVD was based on self-report of high blood pressure, stroke or heart disease of the mother, father or siblings.

We did not adjust for adult health and lifestyle factors, such as diabetes, body mass index (BMI), smoking, alcohol intake and physical activity, as these are likely to be mediators in the relationship between childhood maltreatment and CVD.

Covariates

All covariates were assessed at baseline. Assessment centre corresponds to the 22 assessment centres located across the United Kingdom. Qualification was assessed in categories (College, university or other professional degree; Advance levels/ Advance Subsidiary levels or equivalent; Ordinary levels/ General Certificate of Secondary Educations or equivalent; Certificate of Secondary Educations or equivalent, National Vocational Qualifications; Higher National Diploma, Higher National Certificate or equivalent; Other professional qualification, and None). Smoking status was categorised as never, previous or current smoker. Alcohol intake frequency was assessed by the question "About how often do you drink alcohol?", with response options: daily or almost daily, three or four times a week, once or twice a week, one to three times a month, special occasions only, or never. Depression was defined as a positive answer to either questions: "Have you ever seen a general practitioner (GP) for nerves, anxiety, tension or depression?" or "Have you ever seen a psychiatrist for nerves, anxiety, tension or depression?". Body mass index (BMI) was calculated using weight and height measured at baseline.

Propensity score and weighting procedure

A logistic regression was fitted, separately for men and women, to estimate the propensity score (PS) for responding to the online questionnaire assessing childhood maltreatment. The model included covariates that predicted both responsiveness to the questionnaire and CVD. We then derived stabilised inverse probability weights (IPW), which were used in the substantive models of the associations between childhood maltreatment and CVD outcomes.

The PS was estimated using logistic regression, regressing response to the online questionnaire assessing childhood maltreatment on: age (in 5-year groups), ethnicity,

assessment centre, Townsend deprivation index, qualifications, maternal smoking, smoking status, alcohol intake, depression, use of blood pressure medication, use of cholesterol-lowering medication, SBP, DBP, BMI (in quintiles), and family history of CVD.

The IPW in men had mean 2.0, varying from 1.02 to 32.4, and in women, mean 1.99, ranging from 1.01 to 27.1. Stabilised weights (SW) were calculated by $\mathrm{SW}i = \frac{\mathrm{p}}{\pi i}$ in those who responded to the online questionnaire and $\mathrm{SW}i = \frac{1-\mathrm{p}}{1-\pi i}$ in those who did not respond to the online questionnaire, where p is the probability of responding the online questionnaire without considering the covariates and πi corresponds to the probability of responding to the online questionnaire considering the covariates (Xu, 2010). The mean value of SW for both men and women was 1.0 (standard deviation 0.33 in men and 0.35 in women), with range 0.45 – 9.64 in men and 0.47 – 8.82 in women. Stabilised IPW were used in all analyses using the weighting procedure.

The standardised difference of the covariates before and after weighting is presented in eFigure 5. A covariate is considered balanced if the absolute value of the standardised difference is less than 0.05 standard deviations.

Missing data

There were missing data for the covariates to be used in the PS and adjustment. The highest proportion of missing data was 2.0% (qualification) in the full sample and 1.4% (family history of CVD) in the sample with data for childhood maltreatment.

Complete case analysis (CCA) was shown to be a useful way to handle missing values in covariates for propensity scores (Choi, 2019). Though CCA can introduce bias in the estimates, the amount of bias is likely to be small, given 96.4% of the sample with data on childhood maltreatment had complete data for all covariates (Madley-Dowd, 2019; White, 2010). Therefore, we opted to perform CCA.

References

- Choi J, Dekkers OM, le Cessie S. A comparison of different methods to handle missing data in the context of propensity score analysis. Eur J Epidemiol. 2019;34(1):23-36.
- Madley-Dowd P, Hughes R, Tilling K, Heron J. The proportion of missing data should not be used to guide decisions on multiple imputation. J Clin Epidemiol. 2019;110:63-73.

- White IR, Carlin JB. Bias and efficiency of multiple imputation compared with complete-case analysis for missing covariate values. Stat Med. 2010;29(28):2920-31.
- Xu S, Ross C, Raebel MA, Shetterly S, Blanchette C and Smith D. Use of stabilized inverse propensity scores as weights to directly estimate relative risk and its confidence intervals. *Value Health*. 2010; 13:273-277

eTable 1. Assessment of childhood maltreatment. All sentences referred to "When I was growing up".

Maltreatment	Sentence	Cut-off
Physical abuse	People in my family hit me so hard that it left me with bruises or marks	≥ 1
Sexual abuse	Someone molested me (sexually)	≥ 1
Emotional abuse	I felt that someone in my family hated me	≥ 1
Emotional neglect	I felt loved	≤ 2
Physical neglect	There was someone to take me to the doctor if I needed it	≤ 3

For all sentences, the response options were: 0: never true; 1: rarely true; 2: sometimes true; 3: often true; 4: very often true

eTable 2. Occurrence of cardiovascular disease (CVD) among men and women who responded to the online questionnaire assessing childhood maltreatment

		Men		Women	
CVD	Age	N cases	% (95% CI)	N cases	% (95% CI)
Any source					
Any CVD	All	28,853	42.3 (41.9; 42.7)	28,415	31.9 (31.6; 32.2)
	40-49 y	3,361	22.6 (21.9; 23.3)	4,143	18.6 (18.1; 19.1)
	50-59 y	9,501	39.6 (39.0; 40.3)	10,326	29.9 (29.4; 30.4)
	60+ y	15,894	54.6 (54.1; 55.2)	13,772	43.5 (43.0; 44.1)
Hypertensive disease	All	29,504	43.3 (42.9; 43.6)	25,690	28.9 (28.6; 29.2)
	40-49 y	3,189	25.9 (25.2; 26.6)	2,658	15.3 (14.9; 15.8)
	50-59 y	8,611	42.2 (41.6; 42.8)	8,107	27.9 (27.4; 28.3)
	60+ y	12,926	53.1 (52.5; 53.6)	10,513	39.5 (39.0; 40.1)
Ischemic heart disease	All	5,904	8.7 (8.5; 8.9)	2,666	3.0 (2.9; 3.1)
	40-49 y	302	2.0 (1.8; 2.3)	206	0.9 (0.8; 1.1)
	50-59 y	1,628	6.8 (6.5; 7.1)	760	2.2 (2.0; 2.4)
	60+ y	3,968	13.6 (13.2; 14.0)	1,693	5.3 (5.1; 5.6)
Cerebrovascular disease	All	1,443	2.1 (2.0; 2.2)	1,052	1.2 (1.1; 1.3)
	40-49 y	103	0.7 (0.6; 0.8)	133	0.6 (0.5; 0.7)
	50-59 y	398	1.7 (1.5; 1.8)	365	1.1 (1.0; 1.2)
	60+ y	938	3.2 (3.0; 3.4)	549	1.7 (1.6; 1.9)
Early onset CVD (<50 y)	All	6,800	10.0 (9.8; 10.2)	6,292	7.0 (6.9; 7.2)
	40-49 y	1,835	12.3 (11.8; 12.8)	1,807	8.0 (7.7; 8.4)
	50-59 y	3,018	12.5 (12.1; 13.0)	2,765	8.0 (7.7; 8.2)
	60+ y	1,947	6.7 (6.4; 7.0)	1,720	5.4 (5.2; 5.7)
Medical records and measured blood pressure					
Any CVD	All	19,903	29.2 (28.9; 29.5)	18,806	21.1 (20.9; 21.4)
	40-49 y	2,034	13.7 (13.2; 14.3)	2,628	11.8 (11.4; 12.3)
	50-59 y	6,182	25.9 (25.3; 26.5)	6,544	19.1 (18.7; 19.5)
	60+ y	11,562	39.8 (39.3; 40.4)	9,398	29.9 (29.4; 30.4)
Hypertensive disease	All	23,056	33.8 (33.5; 34.2)	18,814	21.1 (20.9; 21.4)
	40-49 y	2,999	20.1 (19.5; 20.7)	2,407	10.7 (10.3; 11.2)
	50-59 y	8,018	33.3 (32.7; 33.9)	7,165	20.7 (20.2; 21.1)
	60+ y	12,016	41.3 (40.7; 41.8)	9,217	29.0 (28.5; 29.5)
Ischemic heart disease	All	5,344	7.8 (7.6; 8.0)	2,303	2.6 (2.5; 2.7)
	40-49 y	272	1.8 (1.6; 2.0)	172	0.8 (0.7; 0.9)
	50-59 y	1,458	6.1 (5.8; 6.4)	650	1.9 (1.7; 2.0)
	60+ y	3,596	12.4 (12.0; 12.8)	1,473	4.7 (4.4; 4.9)
Cerebrovascular disease	All	928	1.4 (1.3; 1.5)	644	0.7 (0.7; 0.8)
	40-49 y	80	0.5 (0.4; 0.7)	83	0.4 (0.3; 0.5)
	50-59 y	273	1.1 (1.0; 1.3)	226	0.7 (0.6; 0.8)
	60+ y	571	2.0 (1.8; 2.1)	329	1.0 (0.9; 1.2)

CVD: cardiovascular disease

Occurrence of CVD includes prevalent and incident cases

eTable 3. Unadjusted and adjusted association between childhood maltreatment and cardiovascular disease in men and women

Outcome	Childhood maltreatment	Men		Women		p-value for sex interaction
		Unadjusted RR (95% CI)	Adjusted RR (95% CI)	Unadjusted RR (95% CI)	Adjusted RR (95% CI)	
Any cardiovascular disease	Physical abuse	1.02 (1.00; 1.04)	1.06 (1.04; 1.08)	1.03 (1.01; 1.05)	1.11 (1.09; 1.13)	0.008
	Sexual abuse	1.04 (1.01; 1.07)	1.03 (1.00; 1.06)	1.03 (1.00; 1.06)	1.07 (1.04; 1.10)	0.106
	Emotional abuse	1.01 (0.98; 1.03)	1.06 (1.04; 1.09)	1.02 (1.00; 1.04)	1.09 (1.07; 1.12)	0.345
	Emotional neglect	1.04 (1.02; 1.06)	1.05 (1.04; 1.07)	1.06 (1.04; 1.08)	1.08 (1.06; 1.10)	0.069
	Physical neglect	1.14 (1.12; 1.16)	1.08 (1.06; 1.10)	1.16 (1.13; 1.18)	1.07 (1.05; 1.09)	0.354
Hypertensive disease	Physical abuse	1.02 (1.00; 1.04)	1.06 (1.04; 1.09)	1.01 (0.98; 1.04)	1.10 (1.07; 1.13)	0.338
	Sexual abuse	1.02 (0.98; 1.06)	1.01 (0.97; 1.05)	1.00 (0.96; 1.03)	1.04 (1.00; 1.07)	0.421
	Emotional abuse	1.00 (0.97; 1.03)	1.06 (1.03; 1.09)	0.98 (0.95; 1.01)	1.07 (1.04; 1.10)	0.380
	Emotional neglect	1.03 (1.01; 1.05)	1.04 (1.02; 1.07)	1.05 (1.02; 1.08)	1.07 (1.05; 1.10)	0.317
	Physical neglect	1.17 (1.14; 1.19)	1.10 (1.07; 1.12)	1.19 (1.16; 1.23)	1.08 (1.06; 1.11)	0.279
Ischemic heart disease	Physical abuse	1.08 (1.02; 1.15)	1.20 (1.13; 1.27)	1.27 (1.15; 1.40)	1.48 (1.34; 1.63)	<0.001
	Sexual abuse	1.21 (1.09; 1.34)	1.18 (1.07; 1.31)	1.20 (1.07; 1.35)	1.29 (1.15; 1.45)	0.207
	Emotional abuse	1.15 (1.07; 1.24)	1.31 (1.22; 1.41)	1.33 (1.21; 1.47)	1.53 (1.39; 1.69)	0.009
	Emotional neglect	1.14 (1.07; 1.21)	1.18 (1.11; 1.25)	1.37 (1.26; 1.50)	1.41 (1.29; 1.54)	<0.001
	Physical neglect	1.33 (1.25; 1.42)	1.16 (1.09; 1.24)	1.52 (1.39; 1.67)	1.28 (1.17; 1.41)	0.022
Cerebrovascular disease	Physical abuse	0.96 (0.83; 1.10)	1.04 (0.91; 1.19)	1.10 (0.93; 1.30)	1.23 (1.04; 1.46)	0.088
	Sexual abuse	1.05 (0.83; 1.32)	1.03 (0.82; 1.30)	1.28 (1.06; 1.55)	1.34 (1.11; 1.62)	0.058
	Emotional abuse	1.27 (1.09; 1.49)	1.43 (1.23; 1.67)	1.15 (0.98; 1.36)	1.27 (1.07; 1.50)	0.439
	Emotional neglect	1.14 (1.00; 1.30)	1.17 (1.03; 1.33)	1.01 (0.86; 1.18)	1.01 (0.86; 1.19)	0.241
	Physical neglect	1.55 (1.36; 1.77)	1.36 (1.19; 1.55)	1.46 (1.24; 1.71)	1.25 (1.07; 1.47)	0.548

Adjusted for age, Townsend deprivation index, ethnicity, maternal smoking, and family history of cardiovascular disease

[*] p-value corresponds to p-value for sex interaction in the adjusted model

eTable 4. Unadjusted and adjusted association between childhood maltreatment and cardiovascular disease, based on medical records and measured blood pressure, in men and women

Outcome	Childhood maltreatment	Men		Women		p-value for sex interaction
		Unadjusted RR (95% CI)	Adjusted RR (95% CI)	Unadjusted RR (95% CI)	Adjusted RR (95% CI)	
Any cardiovascular disease	Physical abuse	1.02 (1.00; 1.04)	1.07 (1.05; 1.09)	1.04 (1.01; 1.06)	1.12 (1.09; 1.15)	0.012
	Sexual abuse	1.03 (0.99; 1.07)	1.03 (0.99; 1.06)	1.04 (1.00; 1.07)	1.08 (1.05; 1.11)	0.044
	Emotional abuse	1.01 (0.98; 1.03)	1.07 (1.04; 1.09)	1.02 (0.99; 1.05)	1.10 (1.07; 1.13)	0.343
	Emotional neglect	1.03 (1.01; 1.06)	1.05 (1.03; 1.07)	1.07 (1.05; 1.10)	1.09 (1.07; 1.12)	0.022
	Physical neglect	1.14 (1.11; 1.17)	1.08 (1.06; 1.10)	1.17 (1.14; 1.20)	1.08 (1.05; 1.10)	0.272
Hypertensive disease	Physical abuse	1.02 (0.99; 1.04)	1.06 (1.04; 1.09)	1.02 (0.98; 1.05)	1.12 (1.08; 1.16)	0.149
	Sexual abuse	0.99 (0.94; 1.04)	0.98 (0.94; 1.03)	1.00 (0.96; 1.04)	1.05 (1.01; 1.09)	0.078
	Emotional abuse	0.99 (0.96; 1.03)	1.06 (1.03; 1.09)	0.99 (0.95; 1.02)	1.09 (1.05; 1.12)	0.956
	Emotional neglect	1.02 (0.99; 1.05)	1.04 (1.01; 1.07)	1.06 (1.03; 1.09)	1.09 (1.06; 1.12)	0.082
	Physical neglect	1.17 (1.13; 1.20)	1.10 (1.07; 1.13)	1.21 (1.18; 1.25)	1.10 (1.06; 1.13)	0.097
Ischemic heart disease	Physical abuse	1.08 (1.01; 1.15)	1.19 (1.12; 1.27)	1.26 (1.13; 1.40)	1.47 (1.33; 1.63)	<0.001
	Sexual abuse	1.22 (1.09; 1.36)	1.20 (1.08; 1.33)	1.19 (1.05; 1.34)	1.27 (1.12; 1.44)	0.362
	Emotional abuse	1.16 (1.08; 1.26)	1.33 (1.23; 1.43)	1.35 (1.21; 1.49)	1.55 (1.40; 1.72)	0.013
	Emotional neglect	1.14 (1.07; 1.22)	1.18 (1.11; 1.26)	1.34 (1.22; 1.48)	1.39 (1.26; 1.52)	0.003
	Physical neglect	1.32 (1.23; 1.41)	1.15 (1.08; 1.23)	1.48 (1.34; 1.64)	1.25 (1.13; 1.39)	0.066
Cerebrovascular disease	Physical abuse	0.84 (0.71; 0.99)	0.91 (0.77; 1.07)	1.00 (0.81; 1.24)	1.12 (0.90; 1.39)	0.114
	Sexual abuse	1.14 (0.88; 1.48)	1.11 (0.86; 1.44)	1.12 (0.88; 1.43)	1.17 (0.92; 1.50)	0.715
	Emotional abuse	1.25 (1.04; 1.50)	1.37 (1.14; 1.64)	1.10 (0.89; 1.36)	1.21 (0.97; 1.51)	0.410
	Emotional neglect	1.10 (0.95; 1.29)	1.12 (0.96; 1.30)	0.94 (0.77; 1.16)	0.95 (0.77; 1.17)	0.244
	Physical neglect	1.59 (1.36; 1.85)	1.41 (1.21; 1.65)	1.37 (1.12; 1.67)	1.18 (0.97; 1.44)	0.255

Adjusted for age, Townsend deprivation index, ethnicity, maternal smoking, and family history of cardiovascular disease

* p-value corresponds to p-value for sex interaction in the adjusted model

eTable 5. Unadjusted and adjusted association between the number of childhood maltreatment types experienced and cardiovascular disease in men and women

Outcome	Number of childhood maltreatment	Men		Women		p-value for sex interaction
		Unadjusted RR (95% CI)	Adjusted RR (95% CI)	Unadjusted RR (95% CI)	Adjusted RR (95% CI)	
Any cardiovascular disease	1	1.03 (1.01; 1.05)	1.02 (1.01; 1.04)	1.04 (1.02; 1.06)	1.03 (1.01; 1.05)	0.781
	2	1.06 (1.04; 1.09)	1.07 (1.05; 1.09)	1.06 (1.03; 1.09)	1.08 (1.05; 1.11)	
	3	1.06 (1.03; 1.10)	1.09 (1.06; 1.13)	1.08 (1.04; 1.12)	1.12 (1.09; 1.16)	
	4+	1.13 (1.08; 1.17)	1.17 (1.12; 1.22)	1.14 (1.10; 1.19)	1.20 (1.16; 1.24)	
Hypertensive disease	1	1.03 (1.01; 1.05)	1.02 (1.00; 1.05)	1.03 (1.01; 1.06)	1.02 (0.99; 1.04)	0.765
	2	1.06 (1.03; 1.10)	1.07 (1.04; 1.10)	1.02 (0.99; 1.06)	1.04 (1.01; 1.08)	
	3	1.05 (1.01; 1.10)	1.08 (1.04; 1.12)	1.06 (1.01; 1.10)	1.11 (1.06; 1.15)	
	4+	1.15 (1.09; 1.21)	1.19 (1.13; 1.25)	1.13 (1.07; 1.18)	1.19 (1.14; 1.25)	
Ischemic heart disease	1	1.07 (1.01; 1.14)	1.06 (0.99; 1.12)	1.14 (1.03; 1.26)	1.10 (0.99; 1.21)	0.017
	2	1.15 (1.06; 1.25)	1.16 (1.07; 1.26)	1.33 (1.18; 1.50)	1.37 (1.21; 1.54)	
	3	1.24 (1.11; 1.38)	1.31 (1.17; 1.46)	1.49 (1.28; 1.74)	1.60 (1.38; 1.86)	
	4+	1.57 (1.38; 1.79)	1.70 (1.50; 1.93)	1.88 (1.61; 2.19)	2.05 (1.75; 2.39)	
Cerebrovascular disease	1	1.07 (0.94; 1.22)	1.05 (0.92; 1.20)	1.27 (1.08; 1.50)	1.23 (1.04; 1.44)	0.473
	2	1.23 (1.04; 1.45)	1.24 (1.05; 1.46)	1.11 (0.88; 1.38)	1.11 (0.89; 1.39)	
	3	1.20 (0.94; 1.53)	1.25 (0.98; 1.59)	1.17 (0.91; 1.50)	1.22 (0.95; 1.57)	
	4+	1.62 (1.21; 2.17)	1.72 (1.29; 2.29)	1.53 (1.16; 2.02)	1.58 (1.20; 2.09)	

Adjusted for age, Townsend deprivation index, ethnicity, maternal smoking, and family history of cardiovascular disease

[*] p-value corresponds to p-value for sex interaction in the adjusted model

eTable 6. Adjusted association between childhood maltreatment and cardiovascular disease in men and women, by age groups

CVD	Childhood maltreatment	Age group	Men RR (95% CI)	Women RR (95% CI)
Any CVD	Physical abuse	p-value age interaction	0.009	0.830
		40-49y	1.11 (1.05; 1.16)	1.11 (1.05; 1.18)
		50-59y	1.06 (1.03; 1.09)	1.11 (1.07; 1.15)
		60+ y	1.03 (1.01; 1.06)	1.10 (1.07; 1.14)
	Sexual abuse	p-value age interaction	0.059	0.266
		40-49y	1.13 (1.02; 1.24)	1.10 (1.02; 1.18)
		50-59y	1.03 (0.98; 1.08)	1.08 (1.03; 1.12)
		60+ y	1.01 (0.97; 1.04)	1.05 (1.01; 1.08)
	Emotional abuse	p-value age interaction	0.002	0.762
		40-49y	1.11 (1.05; 1.18)	1.10 (1.04; 1.16)
		50-59y	1.08 (1.05; 1.12)	1.10 (1.06; 1.13)
		60+ y	1.02 (0.99; 1.05)	1.09 (1.06; 1.12)
	Emotional neglect	p-value age interaction	0.013	0.012
		40-49y	1.11 (1.05; 1.17)	1.12 (1.06; 1.19)
		50-59y	1.05 (1.02; 1.08)	1.10 (1.07; 1.14)
		60+ y	1.04 (1.02; 1.06)	1.06 (1.03; 1.08)
	Physical neglect	p-value age interaction	0.006	<0.001
		40-49y	1.12 (1.04; 1.20)	1.21 (1.14; 1.29)
		50-59y	1.12 (1.08; 1.15)	1.01 (1.06; 1.14)
		60+ y	1.06 (1.04; 1.08)	1.03 (1.01; 1.06)
Hypertensive disease	Physical abuse	p-value age interaction	0.292	0.373
		40-49y	1.08 (1.01; 1.15)	1.12 (1.04; 1.21)
		50-59y	1.07 (1.03; 1.11)	1.11 (1.06; 1.16)
		60+ y	1.05 (1.02; 1.08)	1.08 (1.04; 1.13)
	Sexual abuse	p-value age interaction	0.544	0.420
		40-49y	1.07 (0.95; 1.21)	1.06 (0.96; 1.17)
		50-59y	0.99 (0.93; 1.06)	1.05 (0.99; 1.11)
		60+ y	1.01 (0.96; 1.05)	1.02 (0.97; 1.07)
	Emotional abuse	p-value age interaction	0.159	0.970
		40-49y	1.07 (0.99; 1.15)	1.05 (0.97; 1.14)
		50-59y	1.09 (1.04; 1.14)	1.07 (1.02; 1.12)
		60+ y	1.04 (1.00; 1.07)	1.07 (1.03; 1.11)
	Emotional neglect	p-value age interaction	0.369	0.032
		40-49y	1.08 (1.01; 1.15)	1.09 (1.02; 1.18)
		50-59y	1.04 (1.00; 1.08)	1.11 (1.06; 1.15)
		60+ y	1.04 (1.01; 1.07)	1.04 (1.01; 1.08)
	Physical neglect	p-value age interaction	0.104	<0.001
		40-49y	1.13 (1.04; 1.23)	1.27 (1.16; 1.39)
		50-59y	1.13 (1.08; 1.18)	1.11 (1.06; 1.16)
		60+ y	1.08 (1.05; 1.11)	1.05 (1.01; 1.08)
		p-value age interaction	0.002	0.030

Ischemic heart disease	Physical abuse	40-49y	1.40 (1.10; 1.78)	1.81 (1.35; 2.43)
		50-59y	1.34 (1.20; 1.50)	1.67 (1.41; 1.97)
		60+ y	1.11 (1.03; 1.20)	1.34 (1.17; 1.54)
	Sexual abuse	p-value age interaction	0.043	0.002
		40-49y	1.59 (1.07; 2.35)	2.14 (1.51; 3.04)
		50-59y	1.31 (1.09; 1.57)	1.29 (1.04; 1.60)
		60+ y	1.10 (0.97; 1.24)	1.16 (1.00; 1.35)
	Emotional abuse	p-value age interaction	0.001	0.332
		40-49y	1.82 (1.41; 2.36)	1.78 (1.31; 2.42)
		50-59y	1.43 (1.26; 1.63)	1.55 (1.32; 1.83)
		60+ y	1.18 (1.08; 1.30)	1.48 (1.30; 1.68)
	Emotional neglect	p-value age interaction	<0.001	0.049
		40-49y	1.73 (1.36; 2.21)	1.73 (1.29; 2.32)
		50-59y	1.28 (1.14; 1.43)	1.53 (1.31; 1.79)
		60+ y	1.10 (1.02; 1.18)	1.33 (1.19; 1.49)
	Physical neglect	p-value age interaction	0.033	0.020
		40-49y	1.38 (1.02; 1.86)	1.69 (1.17; 2.42)
		50-59y	1.28 (1.12; 1.46)	1.46 (1.23; 1.74)
		60+ y	1.11 (1.03; 1.20)	1.19 (1.06; 1.34)
Cerebrovascular disease	Physical abuse	p-value age interaction	0.500	0.277
		40-49y	1.16 (0.74; 1.80)	1.65 (1.11; 2.45)
		50-59y	1.11 (0.88; 1.40)	1.25 (0.95; 1.65)
		60+ y	0.99 (0.83; 1.18)	1.11 (0.86; 1.44)
	Sexual abuse	p-value age interaction	0.813	0.283
		40-49y	1.24 (0.61; 2.51)	0.93 (0.55; 1.59)
		50-59y	1.02 (0.67; 1.53)	1.51 (1.11; 2.04)
		60+ y	1.00 (0.74; 1.35)	1.35 (1.03; 1.76)
	Emotional abuse	p-value age interaction	0.037	0.615
		40-49y	2.45 (1.59; 3.77)	1.31 (0.87; 1.97)
		50-59y	1.37 (1.05; 1.78)	1.38 (1.07; 1.78)
		60+ y	1.30 (1.05; 1.60)	1.20 (0.93; 1.54)
	Emotional neglect	p-value age interaction	0.070	0.042
		40-49y	1.90 (1.27; 2.84)	1.68 (1.12; 2.54)
		50-59y	1.07 (0.85; 1.36)	1.00 (0.78; 1.29)
		60+ y	1.12 (0.95; 1.33)	0.92 (0.73; 1.15)
	Physical neglect	p-value age interaction	0.229	0.755
		40-49y	1.91 (1.15; 3.18)	1.46 (0.91; 2.34)
		50-59y	1.43 (1.10; 1.86)	1.15 (0.86; 1.53)
		60+ y	1.29 (1.10; 1.51)	1.26 (1.02; 1.54)

p-value for interaction test between age groups and childhood maltreatment

Adjusted for age, Townsend deprivation index, ethnicity, maternal smoking, and family history of cardiovascular disease

eTable 7. Adjusted association between the number of childhood maltreatment types and cardiovascular disease in men and women, by age groups

Outcome	N of childhood malt.	Men RR (95% CI)			p-value, age inter.	Women RR (95% CI)			p-value, age inter.
		40-49y	50-59y	60+y		40-49y	50-59y	60+y	
Any CVD					0.001				0.022
	1	1.01 (0.95; 1.07)	1.02 (0.99; 1.05)	1.03 (1.01; 1.05)		1.05 (0.99; 1.12)	1.07 (1.03; 1.10)	1.00 (0.97; 1.02)	
	2	1.10 (1.02; 1.18)	1.07 (1.03; 1.11)	1.06 (1.04; 1.09)		1.11 (1.03; 1.19)	1.12 (1.08; 1.17)	1.05 (1.02; 1.09)	
	3	1.21 (1.11; 1.33)	1.13 (1.08; 1.19)	1.02 (0.98; 1.06)		1.17 (1.07; 1.28)	1.14 (1.08; 1.20)	1.10 (1.05; 1.15)	
	4+	1.25 (1.11; 1.40)	1.18 (1.11; 1.25)	1.12 (1.07; 1.18)		1.28 (1.17; 1.41)	1.20 (1.13; 1.28)	1.17 (1.11; 1.23)	
Hypertensive					0.478				0.033
disease	1	1.04 (0.97; 1.11)	1.02 (0.98; 1.06)	1.03 (1.00; 1.05)		1.02 (0.94; 1.11)	1.06 (1.01; 1.11)	0.99 (0.96; 1.03)	
	2	1.08 (0.99; 1.19)	1.07 (1.01; 1.12)	1.07 (1.03; 1.11)		1.03 (0.93; 1.15)	1.12 (1.06; 1.18)	1.01 (0.96; 1.06)	
	3	1.11 (0.99; 1.25)	1.14 (1.06; 1.22)	1.03 (0.98; 1.09)		1.10 (0.97; 1.25)	1.14 (1.06; 1.22)	1.09 (1.03; 1.16)	
	4+	1.22 (1.05; 1.41)	1.19 (1.10; 1.29)	1.17 (1.10; 1.25)		1.32 (1.16; 1.50)	1.18 (1.09; 1.28)	1.16 (1.08; 1.24)	
IHD					<0.001				0.049
	1	0.82 (0.61; 1.10)	1.17 (1.04; 1.32)	1.04 (0.97; 1.12)		1.49 (1.03; 2.14)	1.17 (0.97; 1.41)	1.04 (0.92; 1.18)	
	2	1.34 (0.94; 1.92)	1.25 (1.07; 1.46)	1.12 (1.02; 1.24)		1.78 (1.17; 2.72)	1.36 (1.09; 1.70)	1.34 (1.15; 1.56)	
	3	2.22 (1.53; 3.22)	1.57 (1.29; 1.91)	1.11 (0.97; 1.28)		1.76 (1.10; 2.82)	1.62 (1.24; 2.12)	1.59 (1.31; 1.93)	
	4+	2.31 (1.52; 3.52)	2.02 (1.63; 2.52)	1.47 (1.25; 1.74)		3.45 (2.19; 5.45)	2.47 (1.92; 3.17)	1.67 (1.33; 2.08)	
Cerebrovascular					0.262				0.761
disease	1	1.28 (0.79; 2.07)	1.01 (0.79; 1.30)	1.05 (0.89; 1.23)		1.48 (0.90; 2.43)	1.36 (1.03; 1.79)	1.14 (0.92; 1.41)	
	2	1.75 (0.99; 3.10)	1.17 (0.86; 1.58)	1.22 (0.99; 1.50)		1.44 (0.85; 2.45)	1.34 (0.95; 1.89)	0.96 (0.69; 1.35)	
	3	2.38 (1.14; 4.97)	1.56 (1.05; 2.32)	0.99 (0.72; 1.36)		1.15 (0.59; 2.27)	1.23 (0.80; 1.90)	1.24 (0.88; 1.73)	
	4+	3.48 (1.68; 7.22)	1.45 (0.88; 2.38)	1.63 (1.10; 2.40)		2.41 (1.29; 4.49)	1.53 (1.00; 2.34)	1.45 (0.95; 2.22)	

CI: confidence interval; CVD: cardiovascular disease; IHD: ischemic heart disease; RR: risk ratio

p-value for interaction test between age groups and childhood maltreatment

Adjusted for age, Townsend deprivation index, ethnicity, maternal smoking, and family history of cardiovascular disease

eTable 8. Adjusted association between childhood trauma and early onset cardiovascular disease, by age groups

Childhood maltreatment	Age group	Men RR (95% CI)	Women RR (95% CI)
Physical abuse	p-value age interaction	0.913	0.810
	40-49y	1.10 (0.99; 1.22)	1.17 (1.05; 1.32)
	50-59y	1.08 (0.99; 1.17)	1.12 (1.01; 1.23)
	60+ y	1.14 (1.01; 1.29)	1.14 (0.98; 1.33)
Sexual abuse	p-value age interaction	0.342	0.816
	40-49y	1.13 (0.93; 1.36)	1.11 (0.96; 1.28)
	50-59y	1.04 (0.90; 1.21)	1.19 (1.06; 1.34)
	60+ y	1.03 (0.83; 1.27)	1.10 (0.93; 1.30)
Emotional abuse	p-value age interaction	0.238	0.817
	40-49y	1.26 (1.12; 1.41)	1.13 (1.00; 1.27)
	50-59y	1.15 (1.04; 1.27)	1.21 (1.10; 1.33)
	60+ y	1.07 (0.92; 1.26)	1.14 (0.98; 1.33)
Emotional neglect	p-value age interaction	0.184	0.010
	40-49y	1.20 (1.08; 1.34)	1.21 (1.09; 1.35)
	50-59y	1.08 (0.99; 1.17)	1.12 (1.03; 1.23)
	60+ y	1.15 (1.02; 1.29)	1.01 (0.88; 1.15)
Physical neglect	p-value age interaction	0.007	<0.001
	40-49y	1.30 (1.14; 1.48)	1.34 (1.18; 1.53)
	50-59y	1.17 (1.05; 1.30)	1.21 (1.09; 1.34)
	60+ y	1.15 (1.02; 1.30)	1.02 (0.89; 1.16)
Number of childhood maltreatment types	p-value age interaction	0.107	0.067
1	40-49y	1.03 (0.91; 1.15)	1.14 (1.01; 1.29)
	50-59y	1.03 (0.94; 1.13)	1.15 (1.04; 1.27)
	60+ y	1.08 (0.96; 1.21)	0.95 (0.83; 1.08)
2	40-49y	1.28 (1.11; 1.48)	1.18 (1.01; 1.37)
	50-59y	1.10 (0.98; 1.23)	1.24 (1.09; 1.40)
	60+ y	1.15 (0.97; 1.35)	0.97 (0.81; 1.16)
3	40-49y	1.29 (1.07; 1.54)	1.26 (1.05; 1.51)
	50-59y	1.16 (0.99; 1.36)	1.26 (1.08; 1.46)
	60+ y	1.08 (0.86; 1.35)	1.02 (0.81; 1.28)
4+	40-49y	1.45 (1.16; 1.81)	1.47 (1.21; 1.78)
	50-59y	1.31 (1.09; 1.57)	1.32 (1.12; 1.57)
	60+ y	1.51 (1.15; 2.00)	1.38 (1.06; 1.79)

Adjusted for age, Townsend deprivation index, ethnicity, maternal smoking, and family history of cardiovascular disease.
Reference group corresponds to non-early onset CVD (i.e. no CVD or CVD occurring ≥ 50 years)

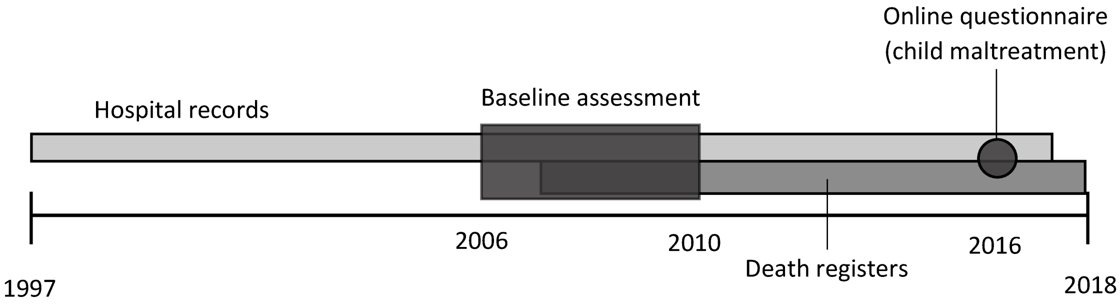

eFigure 1. Timeline for data available

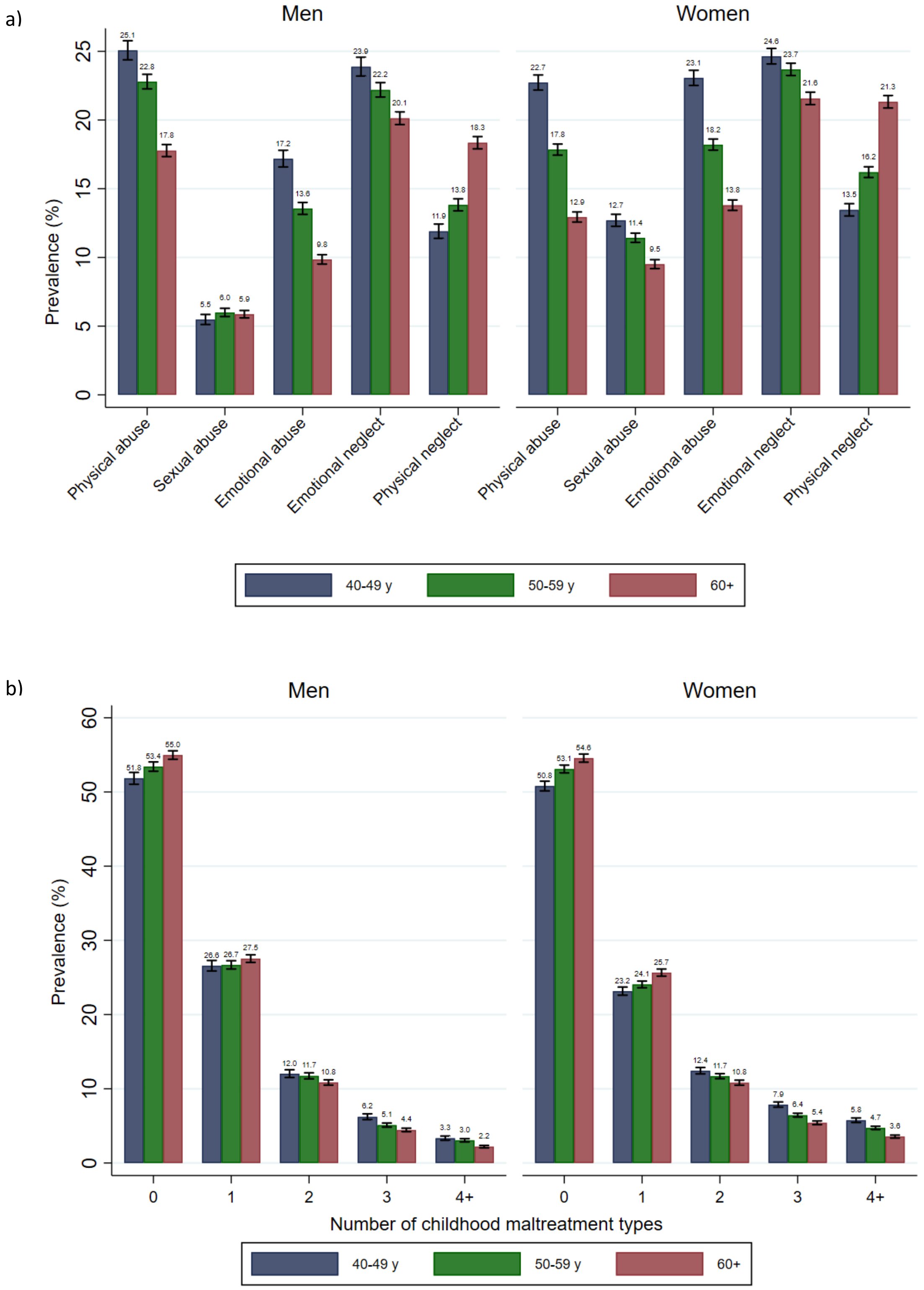

eFigure 2. Prevalence and 95% confidence intervals of types of childhood maltreatment (a) and number of childhood maltreatment types experienced (b) according to age groups in men and women

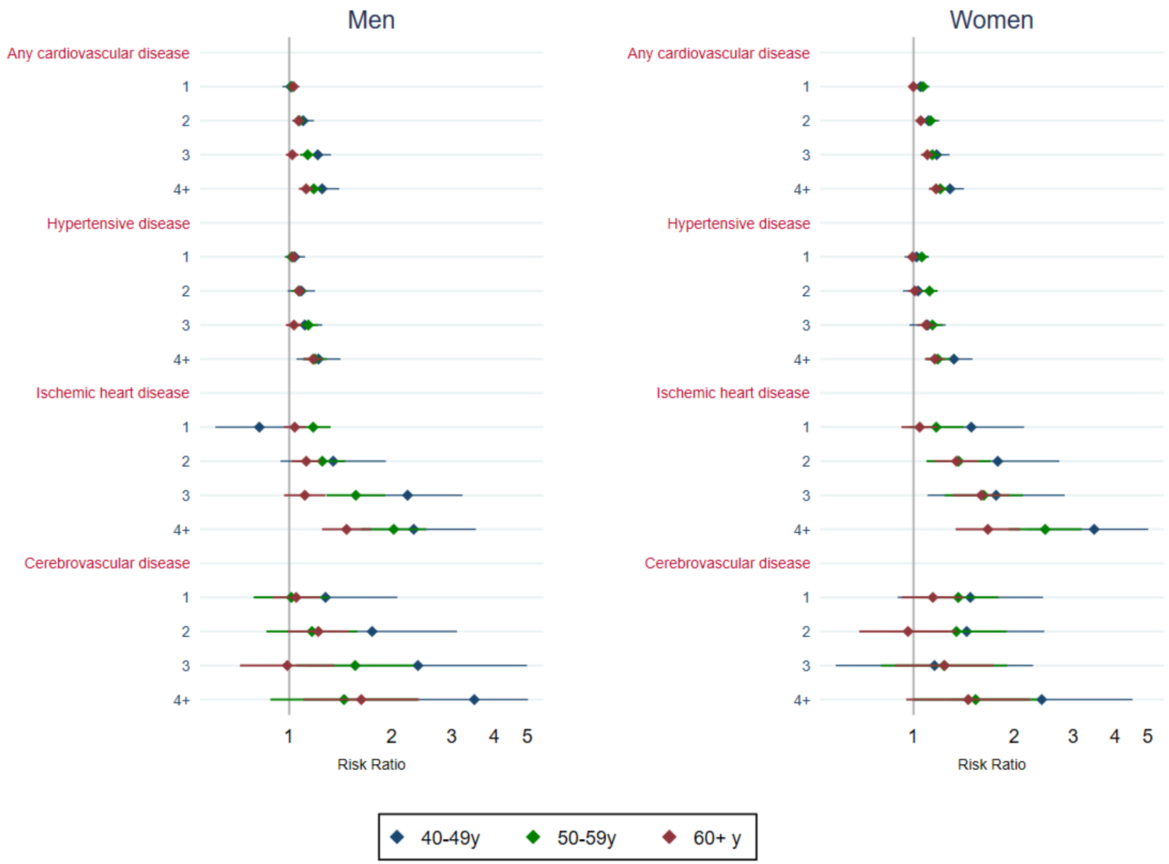

eFigure 3. Adjusted associations (risk ratios and 95% confidence intervals) of number of childhood maltreatment types with cardiovascular disease, according to age groups in men and women.

Adjusted for age, Townsend deprivation index, ethnicity, maternal smoking, and family history of cardiovascular disease

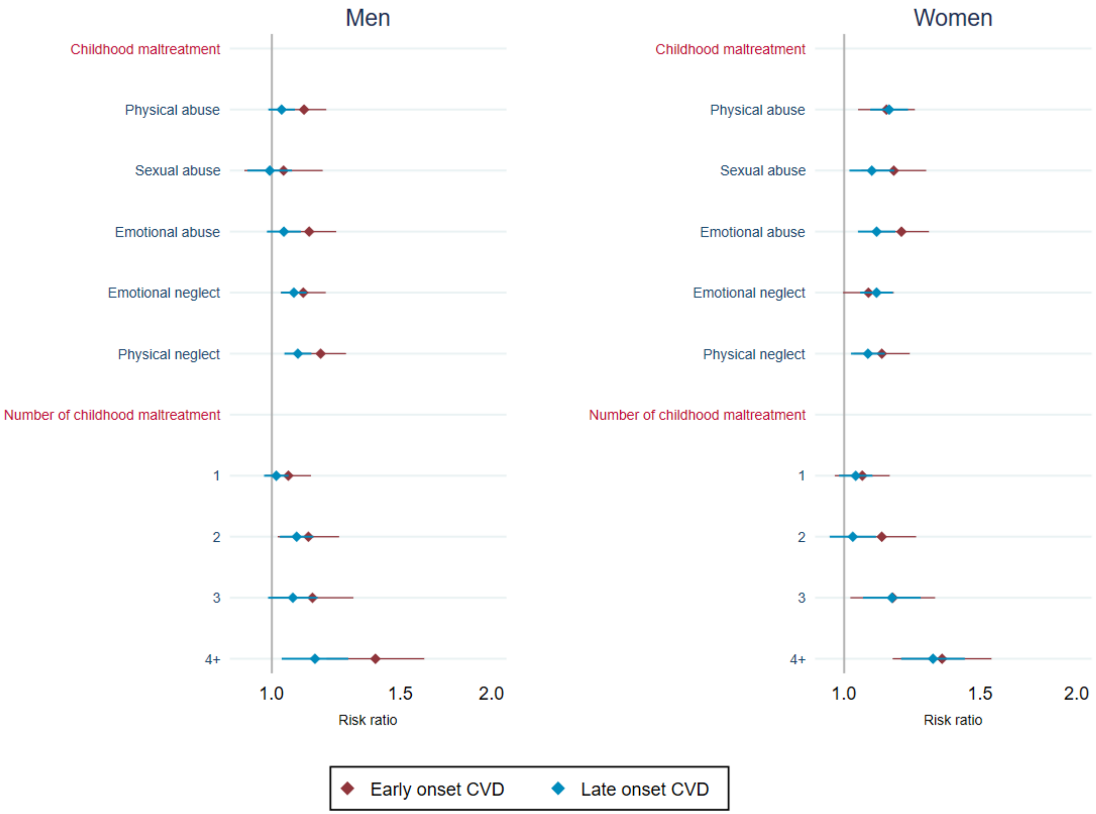

eFigure 4. Adjusted associations (risk ratios and 95% confidence intervals) of childhood maltreatment and number of childhood maltreatment types with early (<50 years) and late onset (≥ 50 years) cardiovascular disease (CVD) in men and women aged 50 years or more.

Adjusted for age, Townsend deprivation index, ethnicity, maternal smoking, and family history of cardiovascular disease

Reference group for both early and late onset CVD is no CVD

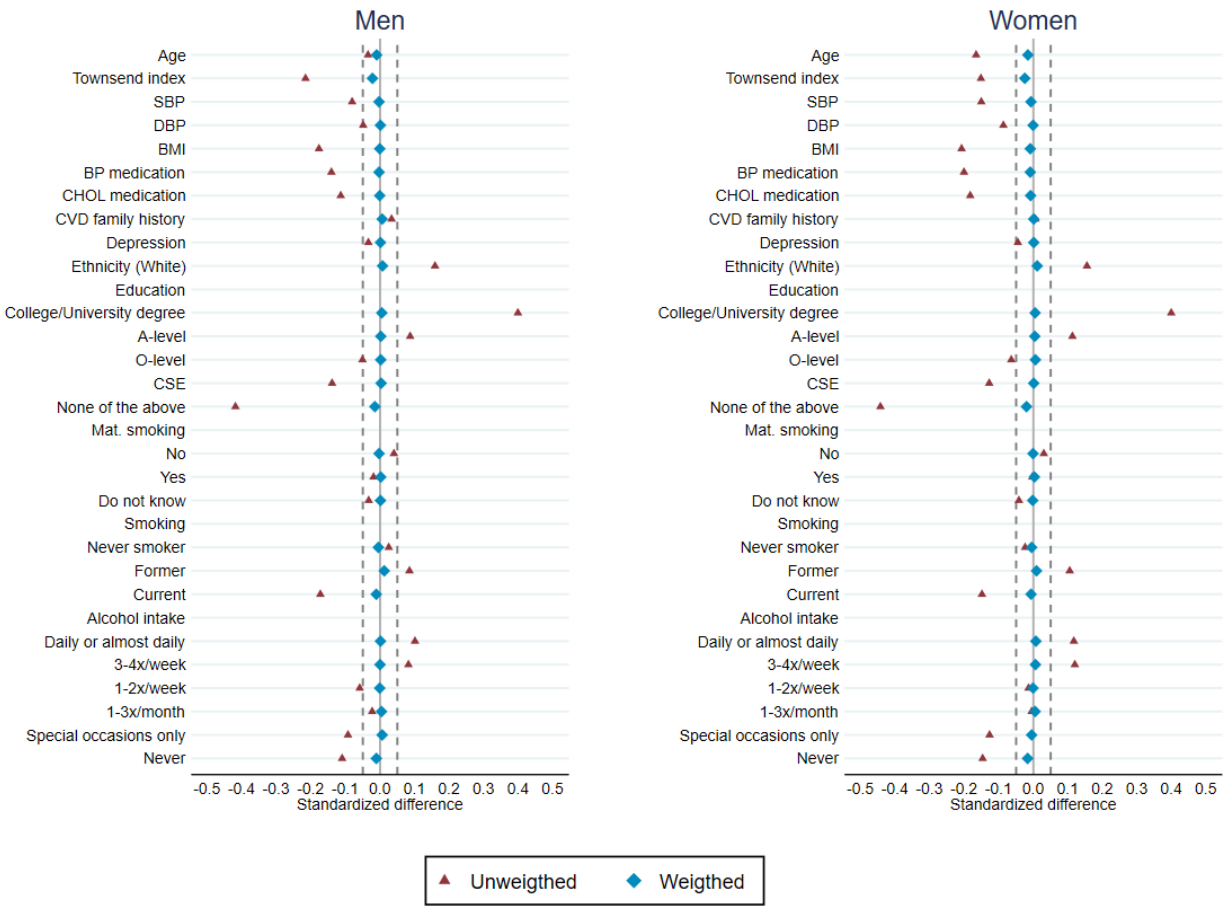

eFigure 5. Balance of covariates (standardised difference) before and after use of inverse probability weighting.

A-level: advanced level; BMI: body mass index; BP: blood pressure; CHOL: cholesterol; CSE: certificate of secondary education; CVD: cardiovascular disease; DBP: diastolic blood pressure; O-level: ordinary level; SBP: systolic blood pressure
